# Exploring the role of unconventional T cells in rheumatoid arthritis

**DOI:** 10.3389/fimmu.2025.1656994

**Published:** 2025-08-20

**Authors:** Tangqing Xu, Hao Cai, Jianye Liu, Xingxing Mao, Yulong Chen, Minhao Chen, Youhua Wang

**Affiliations:** Department of Orthopedics, Affiliated Hospital of Nantong University, Medical School of Nantong University, Nantong, Jiangsu, China

**Keywords:** unconventional T cells, natural killer T (NKT) cells, mucosal-associated invariant T (MAIT) cells, gamma delta T (γδ T) cells, rheumatoid arthritis (RA)

## Abstract

Rheumatoid arthritis (RA) is a chronic autoimmune disorder characterized by sustained synovial inflammation and the gradual destruction of joint structures. Although conventional T cells have historically been viewed as central to RA pathogenesis, increasing attention has recently focused on unconventional T cell subsets, such as natural killer T (NKT) cells, mucosal-associated invariant T (MAIT) cells, and gamma delta T (γδ T) cells. Functioning as a bridge between innate and adaptive immunity, these cells contribute to RA immunopathogenesis by producing cytokines, exerting cytotoxic effects, and interacting with various immune and stromal cells. This review offers a comprehensive analysis of the immunological characteristics and pathogenic roles of unconventional T cell subsets in RA. NKT, MAIT, and γδ T cells contribute to the amplification of inflammatory responses and joint tissue destruction through diverse mechanisms, exhibiting unique tissue tropism and functional plasticity. Recently, novel therapeutic strategies have been developed to target these subsets, including modulation of antigen presentation pathways, inhibition of pro-inflammatory signaling cascades, and reprogramming of cellular functionalities. Advancements in single-cell omics and spatial immune profiling have facilitated the precise identification and characterization of pathogenic unconventional T cell subsets in the RA synovium, thereby paving the way for personalized immunotherapeutic approaches.

## 1 Introduction

Rheumatoid arthritis (RA) is a chronic autoimmune disorder marked by persistent synovial inflammation, pannus formation, and the progressive degradation of cartilage and bone, ultimately leading to joint deformities and functional disability (1, 2). RA affects approximately 0.5% to 1% of the global population, with a disproportionately higher prevalence among females (3). In addition to joint pathology, RA is often associated with systemic complications such as cardiovascular disease, pulmonary involvement, and anemia, all of which further complicate disease management (4). The pathogenesis of RA is now recognized as a multistage process characterized by progressive immune dysregulation, beginning with the loss of peripheral tolerance and culminating in chronic synovitis and tissue degradation (5).

Aberrant immune activation and dysregulation of immune homeostasis are fundamental to the pathogenesis of RA (6, 7). Although conventional T cell subsets, particularly CD4^+^ and CD8^+^ T cells, have long been implicated in RA immunopathology (8–10), recent studies have underscored the critical contributions of unconventional T cells, also referred to as innate-like T cells, including natural killer T (NKT) cells, mucosal-associated invariant T (MAIT) cells, and gamma delta T (γδ T) cells (11, 12). Unconventional T cells, unlike their conventional counterparts, exhibit semi-invariant or restricted T cell receptor (TCR) usage and recognize non-peptide antigens such as lipid metabolites or vitamin B-derived ligands presented by non-classical MHC molecules. These cells are evolutionarily conserved across vertebrates and integrate both innate and adaptive immune features, enabling them to respond rapidly to inflammatory stimuli (13).

Despite increasing recognition of unconventional T cells in immune regulation, most existing literature has examined NKT, MAIT, or γδ T cells in isolation, and often within the broader context of autoimmune diseases such as systemic lupus erythematosus (SLE), multiple sclerosis (MS), and inflammatory bowel disease (IBD) (14–16). These studies collectively suggest that dysregulated unconventional T cell responses may also play critical roles in the pathogenesis of RA. However, a comprehensive understanding of how these three major subsets—NKT cells, MAIT cells, and γδ T cells—interact with the RA microenvironment remains lacking. This review seeks to fill this critical gap by integrating current knowledge on their immunological characteristics, tissue-specific functions, and subset heterogeneity in RA, while also incorporating recent advances from single-cell omics and spatial immune profiling. To our knowledge, no prior work has synthesized these cellular, molecular, and translational insights into a unified framework, making this review a timely and comprehensive resource for both basic and translational immunology in RA. This review will begin by outlining the classification and immunological characteristics of NKT, MAIT, and γδ T cells, then proceed to a detailed discussion of their functional roles in rheumatoid arthritis, explore current and emerging therapeutic strategies targeting these cells, and conclude with perspectives on future research directions.

## 2 Classification and immunological features of unconventional T cells

Unlike conventional T cells that rely on polymorphic MHC molecules to present peptide antigens, unconventional T cells recognize non-peptide ligands—including glycolipids, vitamin B2 metabolites, and phosphoantigens—presented by non-classical MHC class I-like molecules such as CD1d and MR1 (17). Structurally, CD1d presents glycolipids through a hydrophobic groove (A′ and F′ pockets), accommodating lipid tails and exposing the polar head group for recognition by the invariant TCR of iNKT cells (18). In contrast, MR1 binds vitamin B2–derived metabolites like 5-OP-RU via Schiff base formation at lysine 43 (K43), stabilizing MR1 for recognition by MAIT cells, whose semi-invariant TCRs (TRAV1-2–TRAJ33) dock in a fixed orientation (19). These conserved interactions explain the “semi-invariant” nature of unconventional T cell receptors (**Table 1**).

**Table 1:** Structural and functional comparison of unconventional T Cell subsets.

Feature	NKT cells	MAIT cells	γδ T cells
Antigen-presenting molecule	CD1d	MR1	BTN3A1/BTN2A1 (non-classical)
Ligand type	Glycolipids (e.g., α-GalCer)	Vitamin B2 metabolites (e.g., 5-OP-RU)	Phosphoantigens (e.g., HMBPP), stress ligands
Ligand presentation mechanism	Lipid loading in endosomal compartments; lipid tail embedded in groove	Schiff base covalent binding with K43 lysine; microbial metabolite required	Conformational change in BTN3A1/2A1 induced by phosphoantigen binding
TCR configuration	Semi-invariant (Vα24-Jα18/Vβ11 in humans)	Semi-invariant (TRAV1-2/TRAJ33 + TRBV6/20)	Diverse, but Vγ9Vδ2+ dominant in blood
Binding mode	Diagonal docking over CD1d; head group exposed	Fixed angle docking over MR1 pocket	Recognition of BTN conformational changes; no MHC involved
Tissue localization	Liver, adipose, spleen	Liver, gut, lung mucosa	Vδ1^+^: epithelium; Vγ9Vδ2^+^: blood, joints
Functional polarization markers	iNKT1 (T-bet^+^), iNKT2 (GATA3^+^), iNKT17 (RORγt^+^)	MAIT1 (T-bet^+^), MAIT17 (RORγt^+^)	γδT1 (IFN-γ), γδT17 (IL-17)

Moreover, unconventional T cells serve as a functional bridge between innate and adaptive immunity. Possessing pre-programmed effector functions and limited TCR diversity, these cells can mount immune responses within hours—significantly faster than conventional T cells, which require clonal expansion and antigen processing (20). Their innate-like features—including rapid cytokine production and expression of NK markers such as CD161—position them as frontline immune sentinels in barrier tissues such as the liver, gut, and skin (12). A key feature of these cells is their functional plasticity, enabling them to adapt their phenotype in response to environmental cues. Inflammatory cytokines such as IL-12 and IL-18 activate STAT4 and NF-κB signaling, inducing IFN-γ production in NKT and MAIT cells (21). Conversely, IL-23 and IL-1β promote RORγt expression and IL-17 secretion in γδ T and MAIT cells (22). TGF-β and retinoic acid foster regulatory phenotypes like IL-10–producing NKT10 cells (23). Metabolic regulators such as HIF-1α, AhR, and mTOR integrate signals from hypoxia, nutrients, and microbes to fine-tune transcriptional responses (24, 25).

### 2.1 NKT cells

NKT cells constitute a unique subset of lymphocytes that co-express TCRs and natural killer cell markers, functioning as a crucial bridge between innate and adaptive immunity (26). Based on TCR composition and antigen specificity, NKT cells are classified into three major subsets: type I NKT cells (invariant NKT, iNKT), type II NKT cells, and NKT-like cells (27). Type I NKT cells express a semi-invariant TCR—Vα14-Jα18 paired with Vβ8.2 in mice and Vα24-Jα18 paired with Vβ11 in humans—and recognize glycolipid antigens presented by the non-polymorphic CD1d molecule, with α-galactosylceramide (α-GalCer) serving as the prototypical ligand (28). During thymic development, iNKT cells undergo agonist selection through strong TCR engagement with CD1d-expressing double-positive cortical thymocytes, unlike conventional T cells that interact with thymic epithelial cells. This selection induces the expression of promyelocytic leukemia zinc finger (PLZF), a master regulator that programs their innate-like phenotype (25). Subsequent lineage commitment into iNKT1, iNKT2, or iNKT17 subsets is directed by transcription factors T-bet, GATA3, and RORγt, respectively, under the influence of local cytokines (29). In contrast, type II NKT cells exhibit diverse TCR repertoires and are capable of recognizing a broader spectrum of endogenous and microbial-derived lipid antigens, although their precise biological roles remain poorly defined due to the absence of specific surface markers (12). NKT-like cells, although expressing NK-associated markers such as CD161 and CD56, display highly variable TCRs and do not depend on CD1d-mediated antigen presentation (30). Upon activation, NKT cells rapidly secrete a broad array of cytokines, including IFN-γ, IL-4, IL-10, and IL-17, thereby exerting widespread regulatory effects on dendritic cells, B cells, conventional T cells, macrophages, and NK cells (31). Notably, the functional outcomes of NKT cell activation are context-dependent, with cytokine milieu and antigen presentation influencing whether they assume pro-inflammatory or immunoregulatory roles, thereby highlighting their versatility in immune modulation (32, 33). In mice models, iNKT cells are further categorized into functional subsets according to transcription factor expression and cytokine profiles: iNKT1 (IFN-γ^+^, T-bet^+^), iNKT2 (IL-4^+^, GATA3^+^), iNKT17 (IL-17^+^, RORγt^+^), and iNKT10 (IL-10^+^) (34, 35). These subsets exhibit tissue-specific distributions. Hogquist et al. have demonstrated that iNKT1 cells are prevalent in the liver, whereas iNKT10 cells are enriched in adipose tissue, where they may contribute to the regulation of metabolic homeostasis (36). Although similar functional polarization has been proposed in humans, definitive counterparts to murine iNKT1, iNKT2, and iNKT17 subsets have yet to be conclusively identified (37).

### 2.2 MAIT cells

MAIT cells represent a distinct subset of unconventional T lymphocytes, defined by the expression of a semi-invariant TCR. In humans, the canonical MAIT TCR typically comprises the TRAV1–2 gene segment paired with TRAJ33, TRAJ12, or TRAJ20, and is most frequently associated with TCRβ chains TRBV6 or TRBV20 (38). Unlike conventional T cells, which recognize peptide antigens presented by classical MHC molecules, MAIT cells are restricted by the monomorphic MHC class I-related molecule MR1. They recognize riboflavin (vitamin B2) metabolite derivatives synthesized by a broad spectrum of bacteria and fungi, with 5-OP-RU (5-(2-oxopropylideneamino)-6-D-ribityllumazine) being the most potent and well-characterized ligand (39). MAIT cells are highly enriched in mucosal tissues, including the lungs, liver, and gastrointestinal tract, accounting for up to 20–45% of T cells in the liver and 1–10% in the intestinal lamina propria. They also account for 1–10% of circulating T cells in peripheral blood, though this frequency varies with age, sex, and microbiome exposure (40). Phenotypically, MAIT cells display a memory-like and activated phenotype, even under steady-state conditions. They are characterized by high expression of CD161, CD26, and transcription factors such as PLZF, T-bet, and RORγt, reflecting their rapid effector potential and innate-like properties (41). Functionally, MAIT cells can rapidly produce pro-inflammatory cytokines—including IFN-γ, TNF-α, and IL-17—either through TCR-mediated recognition of microbial ligands or via cytokine-driven activation, particularly in response to IL-12, IL-18, and IL-7 (42). In addition to cytokine secretion, MAIT cells also exhibit cytotoxic activity. They release granzyme B and perforin and express surface markers such as CD107a during degranulation, thereby enabling the direct killing of infected or stressed cells (43). Park et al. discovered that MAIT cell migration and tissue homing are regulated by chemokine receptors such as CCR5, CCR6, CCR9, and CXCR6, facilitating their recruitment to mucosal and inflamed tissues (44). MAIT cell development is thymus-dependent and proceeds through three major developmental stages. Their positive selection in the thymus requires MR1 expression and riboflavin-derived ligands from commensal microbes. After thymic exit, microbial exposure in mucosal sites drives peripheral expansion and maturation. This environmental stimulation activates transcriptional programs involving PLZF, T-bet, and RORγt, shaped by IL-7, IL-12, IL-18, and IL-23 signaling (42, 43, 45). This maturation is regulated by commensal microbial exposure—particularly riboflavin-derived ligands—and transcriptional programs involving PLZF, RORγt, and T-bet. These transcriptional programs drive the differentiation of MAIT cells into functionally distinct subsets such as MAIT1 (IFN-γ^+^), MAIT17 (IL-17^+^), and intermediate states like MAIT2, which may function as progenitors or exhibit plasticity during inflammation (44, 46). In addition to classical MAIT1 and MAIT17 subsets, recent studies have identified a functionally distinct population of CD161^-^ MAIT cells that emerges during chronic inflammation. While CD161^+^ MAIT cells exhibit tissue-homing and antimicrobial properties, the CD161^-^ subset displays a more pronounced pro-inflammatory phenotype, characterized by heightened TNF-α and GM-CSF secretion and reduced cytolytic activity (47, 48). This phenotypic shift is thought to result from persistent TCR-independent stimulation, such as chronic exposure to IL-7, IL-15, or inflammatory cytokines in the synovium. Moreover, these cells exhibit altered MR1 responsiveness and may represent a partially exhausted yet pathologically active subset. In RA, the accumulation of CD161^-^ MAIT cells could therefore contribute to sustained inflammation and tissue injury, highlighting the importance of dissecting MAIT cell heterogeneity in both circulation and synovial tissue (49).

### 2.3 γδ T cells

γδ T cells constitute a distinct subset within the T lymphocyte lineage, characterized by expression of a TCR composed of γ and δ chains, in contrast to conventional αβ T cells (50). Functioning at the interface of innate and adaptive immunity, γδ T cells rapidly respond to cellular stress signals and contribute to immune surveillance, microbial defense, and the maintenance of tissue homeostasis. In humans, γδ T cells are primarily classified into two major subsets based on the variable region of the δ chain: Vδ1^+^ and Vδ2^+^ cells (51). Additionally, a less common Vδ3^+^ subset exists, predominantly in the liver (52). Their thymic development involves lineage imprinting determined by TCR usage (e.g., Vδ1^+^ vs Vγ9Vδ2^+^), Notch signals, and cytokines. Vγ9Vδ2^+^ cells—dominant in peripheral blood—are programmed for IL-17 or IFN-γ production and recognize phosphoantigens via BTN3A1/BTN2A1 complexes (53, 54). Vδ1^+^ T cells are predominantly localized in mucosal and epithelial tissues, including the intestine, skin, and lungs. These cells exhibit broad ligand recognition, including CD1d and endothelial protein C receptor (EPCR), and demonstrate pronounced tissue tropism and functional plasticity (55). In contrast, Vδ2^+^ T cells—typically paired with the Vγ9 chain (Vγ9Vδ2^+^)—represent the predominant γδ T cell population in adult peripheral blood (53). These cells recognize small phosphorylated metabolites (phosphoantigens, pAgs), such as HMBPP—a microbial intermediate produced via the non-mevalonate isoprenoid biosynthesis pathway. Binding of phosphoantigens induces conformational changes in BTN3A1, thereby triggering TCR-dependent activation of Vγ9Vδ2^+^ cells (56). Based on cytokine secretion profiles, γδ T cells can be further subdivided into functional subsets: γδT1 cells, which primarily produce IFN-γ and are involved in antiviral and antitumor responses; and γδT17 cells, which secrete IL-17 and contribute to inflammatory processes and tissue remodeling (57). Upon activation, γδ T cells promptly release effector molecules, including IFN-γ, TNF-α, IL-17, perforin, and granzyme B, facilitating direct cytotoxicity against infected or transformed cells (58). Under inflammatory conditions, they may upregulate antigen-presenting molecules (e.g., HLA-DR) and co-stimulatory molecules (e.g., CD80, CD86), acquiring antigen-presenting cell (APC)-like properties that support αβ T cell activation (59). Importantly, γδ T cells exhibit memory-like features. In particular, Vγ9Vδ2^+^ cells can undergo clonal expansion in response to antigenic stimulation, giving rise to long-lived effector memory populations with the capacity for rapid recall responses, enhanced cytokine production, and durable immune protection (58).

## 3 The role of unconventional T cells in RA

### 3.1 Role of NKT cells in RA

**Figure 1:**
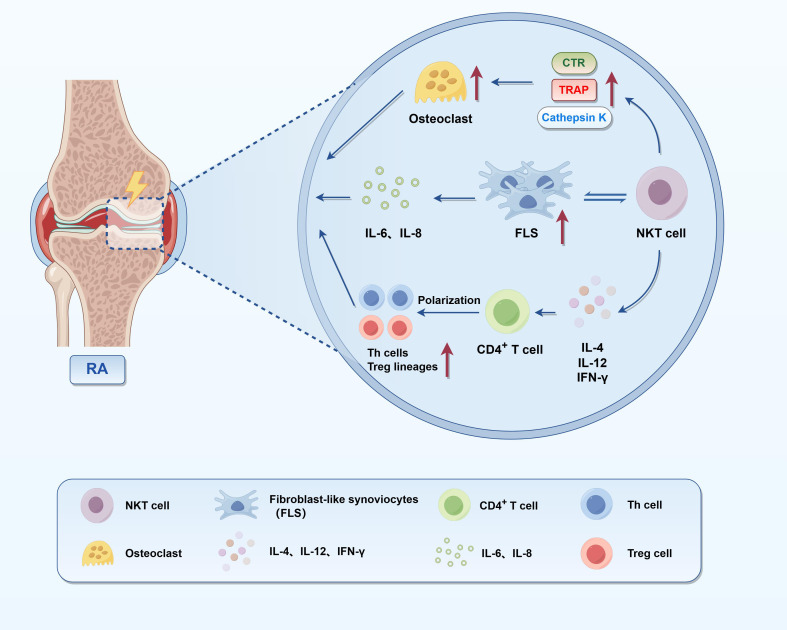
Immunoregulatory and pro-inflammatory roles of NKT cells in RA. This figure illustrates the pathogenic mechanisms of NKT cells in the synovial microenvironment of RA. NKT cells secrete cytokines such as IL-4, IL-12, and IFN-γ, thereby polarizing CD4^+^ T cells into distinct subsets, including Th1, Th2, Th17, and Treg cells. They also interact with FLS, enhancing their proliferation and the production of IL-6 and IL-8, which subsequently stimulate osteoclast precursors and promote the expression of bone-resorptive genes such as TRAP, CTR, and Cathepsin K. Together, these effects underscore the dual role of NKT cells in RA pathogenesis—as regulators of adaptive immunity and as direct contributors to synovial inflammation and joint destruction.

NKT cells, which integrate characteristics of both innate and adaptive immunity, perform multifaceted roles in the immunopathogenesis of RA (**Figure 1**). Linsen et al. observed depleted NKT cells in RA peripheral blood, but functional Th0-like NKT cells persisted in synovial fluid, suggesting they may locally regulate immune responses and contain joint inflammation (60). Functionally, NKT cells exhibit both pro-inflammatory and anti-inflammatory properties. They contribute to inflammatory processes by producing IFN-γ and TNF-α, which enhance macrophage activation and T cell responses. Conversely, they can exert anti-inflammatory effects through the secretion of IL-10 and TGF-β. In RA, NKT cells in synovial fluid predominantly exhibit a pro-inflammatory phenotype, with elevated levels of IFN-γ and reduced IL-10 expression, indicative of a disease-promoting functional shift (61). Coppieters et al. confirmed that beyond cytokine-driven effects, NKT cells also contribute to synovial pathology and joint destruction through multiple effector mechanisms. They interact with fibroblast-like synoviocytes (FLS), promoting their proliferation and stimulating the release of inflammatory mediators such as IL-6 and IL-8. TNF-α released by activated NKT cells binds to TNFR1 on FLSs, activating canonical NF-κB and MAPK signaling cascades (notably p38 and JNK), which leads to increased FLS proliferation and elevated secretion of IL-6 and IL-8. These cytokines further amplify local inflammation and contribute to recruitment of neutrophils and osteoclast precursors. In parallel, TNF-α and IFN-γ upregulate RANKL expression on FLSs and osteoblasts, promoting interaction with RANK on osteoclast progenitors. This triggers TRAF6-dependent signaling and activation of NFATc1, the master transcription factor for osteoclastogenesis, thereby accelerating bone resorption in RA joints (62–65). Furthermore, NKT cells promote osteoclastogenesis by inducing the expression of osteoclast-associated genes—including tartrate-resistant acid phosphatase (TRAP), calcitonin receptor (CTR), and cathepsin K—thereby facilitating bone resorption and joint erosion (62). In addition, NKT cells may influence RA progression through their cytolytic activity. Although the relative proportions of NK and NKT cells may remain unchanged in RA patients, their cytotoxic potential—evidenced by upregulated expression of perforin and granzyme B—is enhanced and correlates with disease severity (63). This suggests that it is not merely their presence, but rather their effector function, that contributes to tissue damage. CD1d, the critical antigen-presenting molecule for NKT cell activation, exists in both membrane-bound and soluble forms. In RA, circulating levels of soluble CD1d (sCD1d) are markedly reduced, impairing peripheral NKT cell activation and IFN-γ production, thereby contributing to immune dysregulation (64). Both experimental and clinical data support the therapeutic activation of NKT cells using glycolipid antigens such as α-GalCer (KRN7000), which can suppress autoimmune inflammation by skewing immune responses toward Th2 or regulatory phenotypes. However, RA patients exhibit heterogeneous responses to α-GalCer stimulation: synovial NKT cells retain partial functionality, whereas peripheral NKT cells often show impaired expansion and diminished IFN-γ secretion (60). Notably, Jin et al. observed that treatment with α-GalCer in mice with collagen-induced arthritis (CIA) models mitigates disease severity, reduces osteoclast activity, and lowers pro-inflammatory cytokine levels—likely via IFN-γ–mediated mechanisms (65). Recent evidence also implicates NKT cells in the regulation of CD4^+^ T cell subset differentiation. NKT-derived cytokines—including IL-4, IL-12, and IFN-γ—significantly influence the polarization of naïve CD4^+^ T cells into Th1, Th2, Th17, or Treg lineages, thereby indirectly shaping the adaptive immune landscape in RA (66). Single-cell transcriptomic profiling of RA synovial tissues has revealed a significant reduction in NKT cell signatures compared to osteoarthritic tissues, underscoring their potential as biomarkers and therapeutic targets within the inflamed synovial microenvironment (67). Overall, NKT cells serve as a crucial link between immune dysregulation, synovial hyperplasia, and bone erosion, and represent promising candidates for immunomodulatory therapies in RA. However, clinical and transcriptomic data reveal a reduction of iNKT cell populations in both peripheral blood and synovial tissues of RA patients. Reduced CD1d expression on antigen-presenting cells may limit iNKT activation and impair IFN-γ production, disrupting the balance between pro-inflammatory and regulatory responses (68, 69). This reduction could result from chronic inflammatory feedback, insufficient survival signals, or persistent antigen exposure that promotes NKT cell exhaustion or egress. Notably, single-cell transcriptomic analyses have failed to detect robust iNKT signatures in inflamed synovium, despite their known capacity for local immunoregulation (70). These findings raise the possibility that iNKT cells exert their protective functions primarily in early RA stages but are numerically and functionally depleted during disease progression. Therefore, their loss may reflect a breakdown of innate regulatory networks, allowing unchecked Th17-driven inflammation and tissue destruction to predominate.

### 3.2 Role of MAIT cells in RA

**Figure 2:**
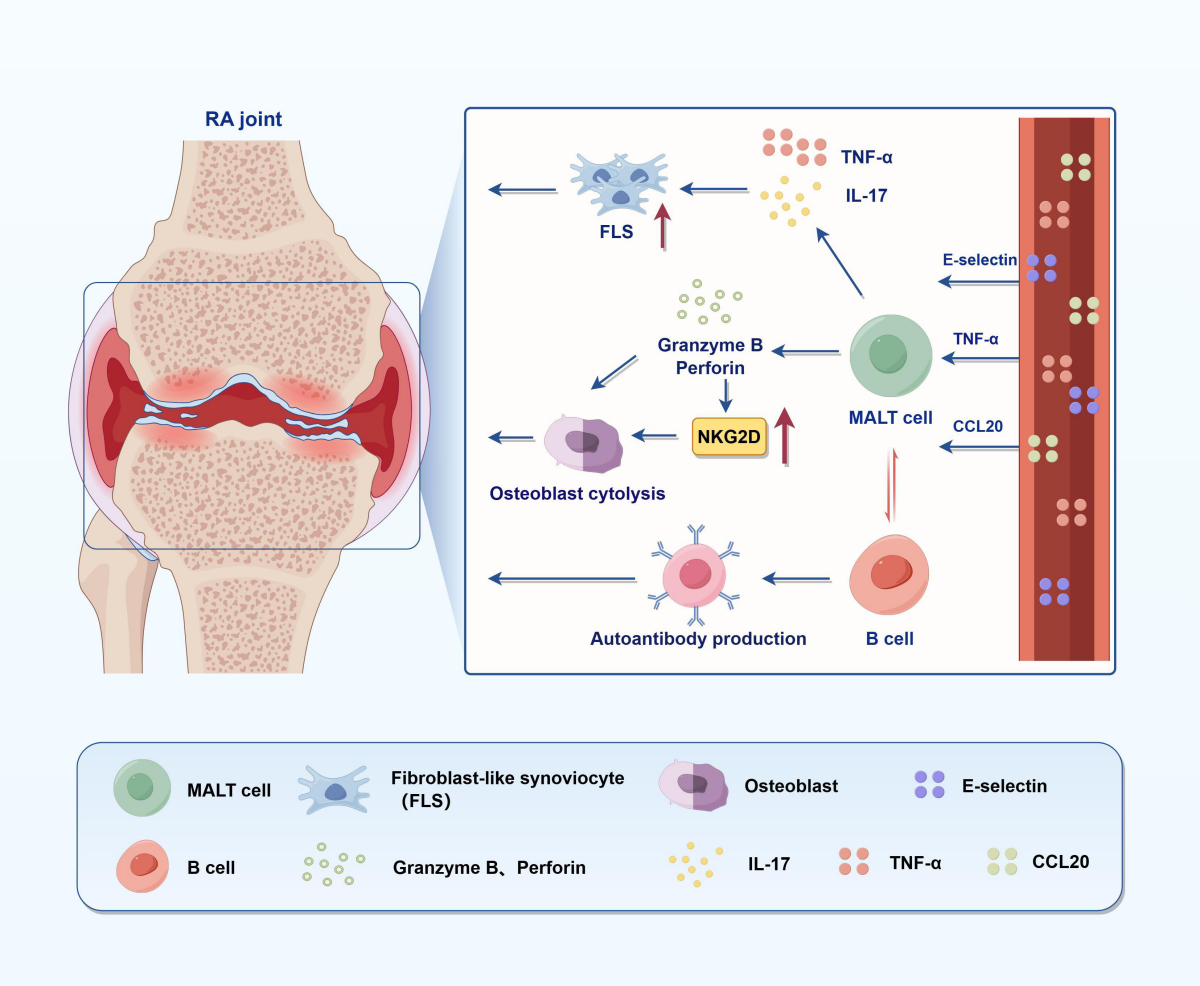
Immunopathogenic role of MAIT cells in RA. This figure illustrates the pathogenic role of MAIT cells in joint inflammation and bone destruction in RA. Upon activation, MAIT cells secrete pro-inflammatory cytokines such as TNF-α and IL-17, which promote the proliferation of FLS, thereby enhancing immune cell recruitment and infiltration. MAIT cells also mediate osteoblast lysis and bone resorption through the release of granzyme B and perforin, and the upregulation of the activating receptor NKG2D. Meanwhile, MAIT cells can interact with B cells to induce autoantibody production, contributing to systemic autoimmunity. Collectively, these mechanisms highlight the multifaceted pathogenic roles of MAIT cells in both local and systemic immune dysregulation in RA.

MAIT cells, defined by their recognition of microbial-derived vitamin B2 metabolites via MR1, are increasingly implicated in RA pathogenesis (**Figure 2**) (19, 71). Zhao et al. demonstrated that, although the frequency of peripheral MAIT cells in RA patients was comparable to that in healthy controls, these cells exhibited distinct phenotypic alterations, including markedly reduced CD161 expression and a skewing toward the CD4^+^ subset (72). Functionally, MAIT cells displayed hyporesponsiveness, as indicated by impaired CD25 and CD69 upregulation upon *E. coli* stimulation, suggestive of chronic activation and functional exhaustion. Importantly, CD161 expression levels were inversely correlated with disease activity, underscoring their potential relevance to RA pathogenesis (72). Furthermore, MAIT cells are enriched in the synovial fluid of RA patients, suggesting tissue recruitment (46). Chiba et al. reported that pro-inflammatory cytokines such as TNF-α and IL-1β upregulate adhesion molecules (e.g., E-selectin) and chemokines (e.g., CCL20), facilitating MAIT cell migration through interactions involving Sialyl Lewis X (SLeX) and CCR6. This supports a model whereby systemic inflammation and vascular activation promote MAIT infiltration into inflamed joints (15, 73). Once within synovial tissues, MAIT cells exhibit heightened activation and cytokine output, notably IL-17 and TNF-α, which contribute to FLS activation and joint damage (46, 71). IL-17 and TNF-α produced by MAIT cells synergistically activate FLSs through NF-κB and STAT3 signaling pathways, leading to increased expression of IL-6, IL-8, and matrix metalloproteinases (MMPs), as well as RANKL, which further drives osteoclastogenesis and cartilage degradation (46, 71, 73). Additionally, MAIT cells mediate osteoblast lysis via granzyme B and perforin release. These cytolytic molecules disrupt mitochondrial integrity, induce cytochrome c release, and activate caspase-dependent apoptotic pathways, thereby contributing to bone loss in RA (71). At the molecular level, MAIT cells promote B cell activation and autoantibody production through direct cell–cell interactions involving CD40L–CD40 signaling and by secreting IL-21, which enhances plasmablast differentiation and IgG class switching (46, 74). They may also exert direct cytotoxicity via granzyme B and perforin pathways, and upregulate NKG2D, further linking them to cytolytic effector functions (44). Notably, in murine CIA models, MR1-deficient mice display attenuated arthritis severity, while adoptive transfer of MAIT cells restores disease intensity—highlighting their effector role independent of TCR signaling and likely mediated via IL-23/IL-1β stimulation (74). These findings align with human data linking MAIT cell-derived IL-17 to joint pathology (73). Furthermore, Li et al. revealed that cross-talk between MAIT cells and B cells may promote autoantibody production. MAIT cells have been shown to increase plasmablasts and Ig production *in vitro*, and may contribute to the humoral autoimmune responses characteristic of RA (46). The dual roles of MAIT cells—protective in some tissues, pathogenic in joints—highlight the complexity of their involvement, which appears dependent on localization, activation context, and disease phase. Although MAIT cells have been implicated in pathogenic inflammation within the joints of RA patients, they also exhibit protective functions in other tissue contexts—particularly at mucosal barriers. In the liver and gut, MAIT cells contribute to epithelial integrity, produce tissue-repair cytokines such as IL-17A and amphiregulin, and help maintain barrier homeostasis following microbial challenge (48). Their rapid response to bacterial metabolites presented via MR1 allows them to limit pathogen dissemination without triggering excessive inflammation. Notably, MAIT cells can produce IL-22 and GM-CSF in the context of commensal-derived antigens, which further supports epithelial regeneration and antimicrobial peptide production (75). In models of intestinal injury (e.g., DSS-induced colitis), MAIT cells were shown to limit barrier breakdown and accelerate recovery through cytokine-driven repair programs, a function partly dependent on IL-17A signaling (76). Similarly, in lung infection models, MAIT cells facilitated pathogen clearance while promoting epithelial regeneration—highlighting their role in host protection without overt tissue damage (77). Taken together, these findings suggest that MAIT cell functional polarization is tissue- and context-dependent. While they may adopt a pathogenic phenotype in inflamed joints—particularly under chronic IL-7/IL-15 stimulation or microbial dysbiosis—they retain the capacity for immunoregulation and tissue protection in non-joint sites. Understanding the signals that drive this dichotomy is critical for therapeutic targeting.

### 3.3 Role of γδ T cells in RA

**Figure 3:**
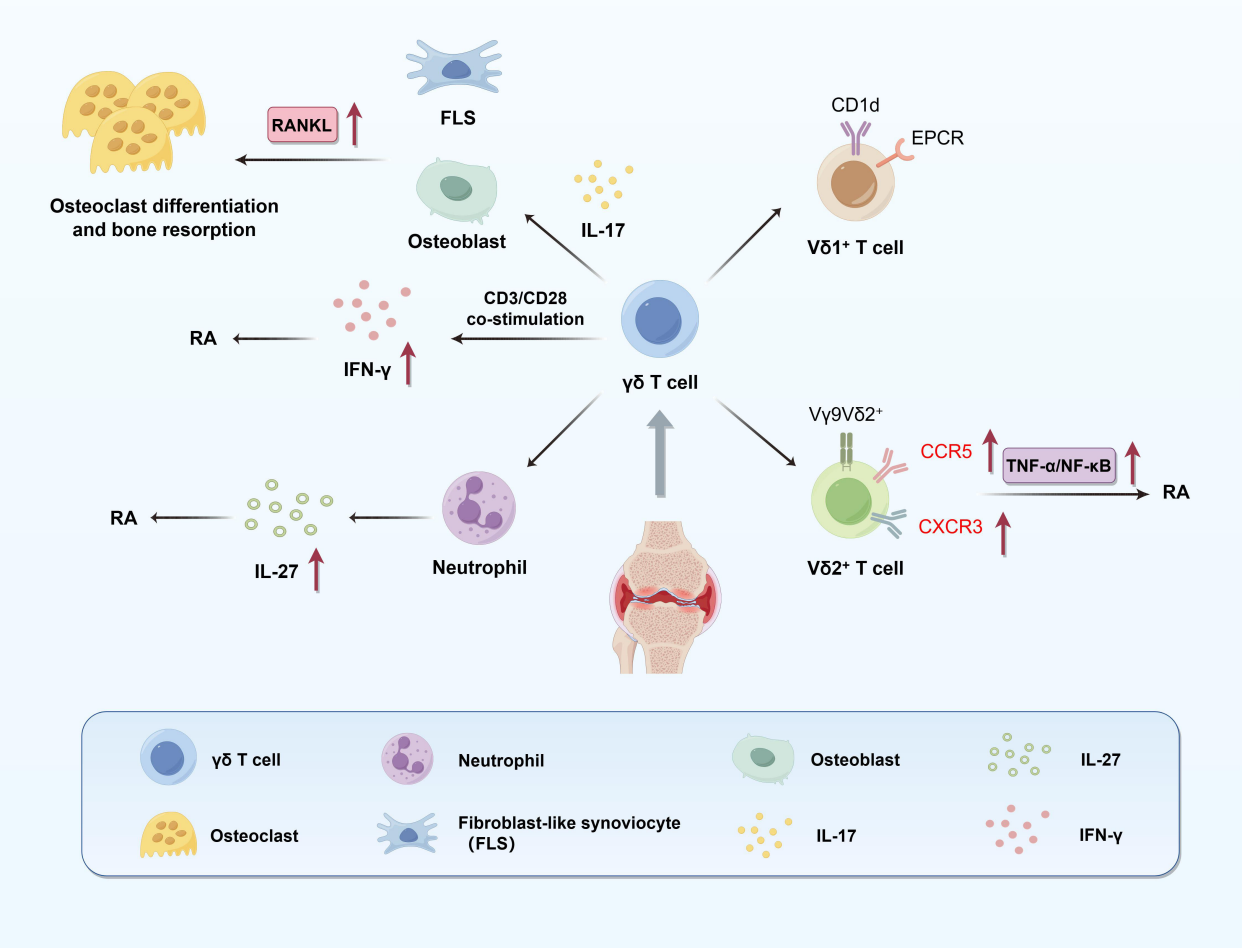
Pathogenic roles of γδ T cells in RA. This figure illustrates the multifaceted contributions of γδ T cells to synovial inflammation and bone destruction in RA. Distinct subsets of γδ T cells play divergent roles: Vδ1^+^ T cells engage in antigen presentation via CD1d and EPCR, while Vγ9Vδ2^+^ T cells promote IL-17 production, which induces RANKL expression in FLS and osteoblasts, thereby facilitating osteoclast differentiation and bone resorption. Vδ2^+^ T cells also upregulate chemokine receptors CCR5 and CXCR3 through the TNF-α/NF-κB pathway, enhancing their recruitment into inflamed joints. Upon CD3/CD28 co-stimulation, γδ T cells secrete IFN-γ, which can counteract bone resorption, highlighting their context-dependent regulatory potential. Additionally, γδ T cells influence neutrophil function; their depletion enhances neutrophil-derived IL-27 production, which negatively regulates RA severity. These mechanisms underscore the dual inflammatory and regulatory roles of γδ T cells in RA pathogenesis.

γδ T cells are a critical subset of unconventional T lymphocytes that bridge innate and adaptive immunity and have been identified as key regulators in the multifactorial pathology of RA (**Figure 3**). However, the reported frequency of γδ T cells in both peripheral blood and synovial fluid remains controversial. While some studies report increased levels, others observe reductions in blood, possibly due to migration into inflamed joints (78–80). These discrepancies likely stem from methodological differences (e.g., flow cytometry panels, subset gating), patient heterogeneity (e.g., disease stage, serostatus), tissue compartment analyzed (blood vs. synovium), and treatment exposure (80–84). This variability highlights the need for standardized protocols and subset-specific analysis to clarify the role of γδ T cells in RA pathogenesis. Although they do not constitute the predominant T cell population in synovial tissue, γδ T cells exert disproportionate influence due to their rapid cytokine secretion, antigen-presenting capabilities, and extensive interactions with other immune cells—playing a potent “small-in-number, strong-in-function” role in the RA inflammatory microenvironment (81). Distinct γδ T cell subsets exert different functions in RA. Vδ1^+^ T cells are mainly distributed in epithelial and mucosal tissues and may possess immunoregulatory properties in RA (82). In contrast, Vδ2^+^ T cells are markedly expanded in peripheral blood and synovial compartments of RA patients, displaying a highly pro-inflammatory phenotype and producing large amounts of IL-17, IFN-γ, and TNF-α, all of which contribute to synovitis and bone erosion (80, 81, 83). γδ T cells promote bone destruction through multiple mechanisms. IL-17 secreted by γδ T cells induces RANKL expression on synovial fibroblasts and osteoblasts. In addition, TNF-α released by γδ or NKT cells binds to TNFR1 on FLSs and osteoblasts, activating the canonical NF-κB and p38 MAPK signaling pathways. These pathways upregulate RANKL transcription via nuclear RelA (p65) translocation, enhancing osteoclastogenic potential and contributing to bone resorption in RA (65, 80). However, under certain conditions, such as CD3/CD28 co-stimulation, γδ T cells produce high levels of IFN-γ, which can inhibit osteoclastogenesis, indicating that γδ T cells may possess environmentally dependent protective effects (59). The migratory potential of γδ T cells is another crucial factor in RA pathogenesis. Mo et al. showed that Vδ2^+^ T cells in RA patients express elevated levels of chemokine receptors CCR5 and CXCR3, upregulated via the TNF-α/NF-κB pathway. Specifically, TNF-α stimulation activates the canonical NF-κB cascade in Vδ2^+^ cells through TNFR1, leading to IκBα degradation and RelA (p65) nuclear translocation, which promotes CCR5 and CXCR3 gene transcription. Anti-TNF-α therapies can downregulate these receptors and partially restore peripheral γδ T cell levels, highlighting their potential as therapeutic targets (80). Beyond classic inflammatory pathways, γδ T cells are closely associated with neutrophil function. Bouchareychas et al. demonstrated that γδ T cells regulate the production of IL-27 in neutrophils. Blockade of γδ T cells leads to increased IL-27 production, which in turn suppresses IL-23-induced arthritis. Blockade of γδ T cells leads to increased IL-27 production, which in turn suppresses IL-23-induced arthritis. Mechanistically, IL-27 inhibits STAT3 phosphorylation in IL-23-responsive cells such as Th17 and γδ T17 cells, reducing IL-17 production and limiting downstream inflammatory amplification. This establishes a negative feedback loop between γδ T cells, neutrophils, and IL-27 (83, 85). In addition, γδ T cells exhibit memory-like immune features. For instance, Vγ9Vδ2^+^ T cells in RA can undergo clonal expansion and respond rapidly to antigen re-stimulation, releasing high levels of inflammatory cytokines. This property sustains chronic inflammation and plays a pivotal role in the persistent phase of RA (83). Interestingly, γδ T cells may also exert regulatory or protective functions. For instance, Su et al. elucidated that treatment of RA patients with ^99^Tc-methylene diphosphonate (^99^Tc-MDP) significantly increases γδ T cells and Tregs, alongside decreased TNF-α and IL-6 levels and elevated TGF-β expression, indicating a potential role in promoting immune tolerance under specific conditions (84). Moreover, in rat adjuvant arthritis models, depletion of γδ T cells exacerbates joint destruction, further supporting their stage-dependent dual role in RA (86). In RA synovial fluid, γδ T cells also exhibit activation-dependent phenotypic changes, including downregulation of CD16 (FcγRIII) and upregulation of HLA-DR, suggesting a role in antigen presentation and further amplification of immune responses (81). In summary, γδ T cells play multifaceted roles in RA, functioning as both drivers of pathogenic inflammation and potential immune regulators depending on microenvironmental cues (**Table 2**).

**Table 2:** Comparative summary of MAIT, NKT, and γδ T cells in the synovial inflammatory cascade of RA.

Feature/Function	NKT Cells	MAIT Cells	γδ T Cells
Key Cytokines Produced	TNF-α, IFN-γ, IL-4	IL-17, TNF-α, IFN-γ, IL-21	IL-17, IFN-γ, GM-CSF
Cytotoxic Molecules	Moderate granzyme/perforin	Granzyme B, perforin (targeting osteoblasts)	Granzyme B (minor), perforin (rare)
Activated By	CD1d-presented lipids, cytokines	MR1-ligands, IL-12 + IL-18, microbial vitamin B metabolites	IL-1β, IL-23, TCR ligands, synovial stress
Main Target Cells in Synovium	FLSs, osteoblasts	FLSs, osteoblasts, B cells	FLSs, neutrophils, osteoclast precursors
Pro-inflammatory Actions	Stimulates FLS proliferation, RANKL expression, and IL-6 release	Induces FLS activation, osteoblast lysis, and B cell help	Promotes osteoclastogenesis via RANKL induction and IL-17; recruits neutrophils
Regulatory Potential	Dual role: IL-4-producing NKT may restrain synovial inflammation	Possible suppression via PD-1 expression in chronic phase	Induces IL-27 via neutrophils; IFN-γ^+^ subsets inhibit osteoclasts
Migration Signals	CXCR3, CXCR6	High CXCR6, CCR6 (dependent on IL-12/IL-18)	High CCR5, CXCR3 (via TNF-α/NF-κB pathway)
Role in Autoantibody Production	Indirect; may shape cytokine milieu favoring B cell activation	Direct via CD40L and IL-21 signals to B cells	Indirect (via neutrophil–B cell crosstalk)
Overall Contribution to RA	Bridges innate and adaptive inflammation; context-dependent effects	Amplifies inflammation and B cell autoimmunity	Major driver of early joint inflammation and bone erosion

## 4 Therapeutic targeting of unconventional T cells in RA

The pathogenic involvement of unconventional T cells in RA highlights their therapeutic relevance, but also underscores the need for molecular precision, translational feasibility, and patient-specific immune profiling. Among these subsets, IL-17–producing Vγ9Vδ2^+^ γδ T cells, defined by expression of the TRGV9 and TRDV2 gene segments, have emerged as critical mediators of synovial inflammation and bone erosion (80, 83). Targeted strategies include monoclonal antibodies against Vδ2 or Vγ9 TCR chains, as well as inhibitors of the BTN3A1–phosphoantigen interaction, which is essential for γδ TCR activation (56). Additionally, IL-17/IL-23 axis blockade using agents such as secukinumab or guselkumab has shown efficacy in suppressing γδ T cell–mediated inflammation, though clinical outcomes in RA remain modest due to cytokine redundancy. However, achieving subset-specific suppression without compromising protective anti-infective γδ T cell functions remains a major translational challenge. Broad suppression of IL-17 may impair mucosal defenses, while BTN-targeted approaches risk interfering with other innate-like cells (74, 80). Moreover, redundancy with Th17 cells and MAIT cells producing similar cytokines complicates durable disease control. Although the IL-17/IL-23 axis is mechanistically implicated in RA pathogenesis, clinical trials targeting these cytokines have yielded limited efficacy. For instance, IL-23 inhibitors such as guselkumab and dual IL-12/23 blockade with ustekinumab have not demonstrated superior clinical benefit compared to TNF inhibitors (87). These outcomes highlight the complexity of cytokine redundancy and immune compensation in chronic autoimmune settings. In contrast, CTLA-4–Ig fusion protein (abatacept), which modulates T cell costimulation, has shown sustained efficacy in RA and is widely used in clinical practice (88). These observations underscore the need for therapeutic strategies that account for immune plasticity and the overlapping functions of unconventional T cells within the broader inflammatory network. iNKT cells, characterized by the Vα24-Jα18/Vβ11 TCR and restricted by CD1d, are immunologically dualistic—capable of pro-inflammatory or regulatory roles (26). Activation with α-GalCer analogs such as OCH or C20:2 promotes Th2-skewing cytokine responses, attenuating synovitis (39). Conversely, blockade of CD1d–lipid interactions can prevent pro-inflammatory iNKT activation in patients with heightened NKT effector profiles (64). Restoration of soluble CD1d (sCD1d) has also shown potential to revive IFN-γ production from peripheral NKT cells (64). However, the functional plasticity of iNKT cells, their widespread tissue distribution, and the ubiquity of CD1d expression pose challenges for cell-specific modulation. Overactivation may induce systemic immune activation or inadvertently dampen beneficial NKT-mediated regulatory responses. MAIT cells, defined by a semi-invariant TCR composed of TRAV1–2 and TRAJ33/12/20, recognize bacterial riboflavin metabolites presented by MR1 (39). Targeted approaches include MR1 antagonists that block microbial ligand presentation and experimental TCR-blocking biologics that disrupt MAIT activation (43). Moreover, modulation of the gut microbiome—via antibiotics, probiotics, or microbial metabolite inhibitors—has been proposed to normalize MAIT cell activity by suppressing pathogenic riboflavin-producing bacteria (e.g., via ribD/ribE pathways) (46). Yet, MAIT cells are essential for mucosal homeostasis and antimicrobial defense; systemic suppression risks infections and gut barrier dysfunction (39, 43). Beyond therapeutic targeting, biomarker-guided patient stratification is increasingly recognized as critical. Single-cell RNA sequencing and TCR repertoire profiling have identified hyperexpanded γδ T or MAIT clones with pathogenic gene signatures (67). For example, Vγ9Vδ2^+^ expansion and high IL-17A expression may predict benefit from IL-17 or BTN3A1 blockade, whereas exhausted CD161^low MAIT cells with impaired cytokine response may benefit from microbiota-based modulation (46, 67). NKT cell deficiency or altered CD1d expression profiles could guide use of α-GalCer analogs or sCD1d restoration (26, 64). Such immune signatures enable development of predictive biomarkers for therapy response, allowing precision immunotherapy tailored to dominant unconventional T cell circuits in individual patients. Nonetheless, the inherent plasticity and redundancy among unconventional T cells, and their interface with adaptive immunity, demand combinatorial strategies that target multiple axes while preserving immune homeostasis. Rational co-targeting of unconventional T cells and immune checkpoints (e.g., PD-1, CTLA-4) has shown synergistic effects in restoring regulatory balance and effector control (67).

## 5 Conclusions and prospectives

Unconventional T cells—including NKT cells, MAIT cells, and γδ T cells—are now recognized as critical immunological regulators and effectors in the pathogenesis of RA. Through the secretion of pro-inflammatory cytokines such as IFN-γ, TNF-α, and IL-17, and through direct interaction with synoviocytes, dendritic cells, B cells, and osteoclast precursors, these cells modulate both innate and adaptive immune responses within the RA microenvironment. Notably, the tissue migratory behavior of these unconventional T cells may be shaped by factors such as disease stage, local microenvironment, and subset heterogeneity, and their distribution between peripheral blood and synovial tissue remains incompletely understood and subject to ongoing debate. Despite growing mechanistic insights, critical questions remain. The molecular signals that dictate the phenotypic polarization and effector specialization of unconventional T cells in RA remain poorly defined. The balance between their regulatory and pathogenic roles is context-dependent, and likely modulated by local cytokine milieus, antigenic stimuli, and cell–cell interactions.

To advance our understanding of unconventional T cells in RA, future research should integrate mechanistic, technological, and translational efforts. At the mechanistic level, it remains essential to dissect the molecular cues that govern the activation, differentiation, and plasticity of NKT, MAIT, and γδ T cell subsets within the RA microenvironment. Particular attention should be given to how local cytokine signals—such as IL-23, IL-12, TGF-β, and IL-1β—and metabolic mediators like hypoxia and short-chain fatty acids influence transcriptional regulators such as PLZF, RORγt, and T-bet. These pathways shape the balance between pro-inflammatory and regulatory phenotypes and determine the extent of tissue infiltration and pathogenicity. Elucidating how CD1d and MR1-mediated antigen presentation orchestrates effector function and memory formation will also be crucial in understanding tissue-specific immunopathology. Parallel to this, technological advancements are revolutionizing the study of immune cell heterogeneity. The integration of single-cell RNA sequencing, spatial transcriptomics, high-dimensional cytometry, and TCR repertoire profiling allows for unprecedented resolution in identifying unconventional T cell subsets, mapping their clonal relationships, and determining their spatial localization in the inflamed synovium. When combined with microbiome and metabolome profiling, these tools can elucidate how environmental and microbial factors shape unconventional T cell states across different stages of RA progression. Such multi-omic approaches are poised to uncover novel biomarkers and therapeutic targets. From a translational standpoint, these mechanistic and technological insights must be harnessed to develop precision immunotherapies. Therapeutic strategies could include the expansion or adoptive transfer of regulatory subsets such as iNKT10 or MAIT1 cells, or the targeted suppression of IL-17–producing γδ T cells using monoclonal antibodies or chemokine receptor antagonists (e.g., CCR5, CXCR3). Pharmacological modulation of CD1d and MR1 antigen presentation may further allow fine-tuning of pathogenic T cell activation. Additionally, co-targeting unconventional T cells alongside classical immune checkpoints (e.g., PD-1, CTLA-4) may yield synergistic benefits. Given the emerging role of the microbiota in regulating MAIT and γδ T cell responses, gut-directed interventions—including probiotics or microbial metabolite mimetics—could serve as indirect immunomodulatory approaches. Together, these directions provide a comprehensive roadmap toward harnessing unconventional T cells for next-generation therapies in RA.
